# Efficacy of pan-computed tomography scan in trauma patients presenting in the emergency department of a tertiary care center: an observational study

**DOI:** 10.1097/MS9.0000000000005001

**Published:** 2026-04-17

**Authors:** Subhash Regmi, Bishwa Bandhu Niraula, Pranodan Poudel, Prashanna Dip Karki, Saroj Dahal, Paras Khakurel, Ishor Pradhan

**Affiliations:** Department of Orthopedics and Trauma Surgery, B&B Hospital, Gwarko, Nepal

**Keywords:** diagnostic yield, emergency department, injury severity score, pan-CT, trauma

## Abstract

**Background:**

The use of pan-computed tomography (pan-CT) in trauma care has gained widespread popularity due to its ability to identify life-threatening multi-system injuries. However, concerns remain regarding its liberal use, radiation exposure, and cost-effectiveness, especially in the absence of clear clinical indications.

**Methods:**

This cross-sectional analytical study was conducted at a tertiary care center from May 2023 to April 2025. Records of 313 trauma patients who underwent pan-CT in the emergency department were analyzed. Pan-CT scans were divided into five anatomical zones, and findings were classified as major or minor injuries. Injury Severity Score (ISS) was used to stratify patients into ISS <16 and **≥**16 groups. Chi-square test and binary logistic regression were used for analysis.

**Results:**

Among 313 patients, 257 (82.1%) had positive findings in at least one body region, 166 (53.03%) in two or more regions, and 56 (17.89%) in three or more regions. Emergency interventions were required in 14.4% of patients, and 10.9% underwent additional contrast-enhanced scans. Patients with ISS **≥**16 had significantly higher positivity rates in the head (OR = 2.55, 95% CI 1.61–4.03, *P* < 0.001) and chest (OR = 1.96, 95% CI 1.24–3.11, *P* = 0.004) regions. However, only 14% underwent emergency procedures despite the high positivity rate.

**Conclusion:**

Pan-CT scans demonstrated high positivity rates, especially in patients with ISS >16 and injuries to the head and chest. While effective in detecting injuries, the clinical utility must be balanced with judicious use, and further studies are warranted to establish standardized indications for pan-CT in trauma care.

## Introduction

The availability of computed tomography (CT) scans has revolutionized trauma care management in the emergency department^[^1^]^. Obtaining a pan-CT scan, i.e., CT-scan of head, neck, chest, abdomen, and pelvis, helps in early identification of life-threatening multi-system injuries which can be missed or delayed in following the conventional imaging strategy, i.e., obtaining selective CT scans based on initial evaluation^[^2–4^]^. This resulted in liberal use of obtaining pan-CT scan in the emergency department of many centers.

Studies have shown that there is no significant difference in 30-day mortality in trauma patients who had pan-CT scan compared to those who underwent conventional imaging strategy^[^5,6^]^. In addition, liberal use of obtaining pan-CT scan in the emergency department increases the burden of radiation exposure and treatment costs to the patients^[^7^]^. Literature lacks enough studies evaluating the effectiveness of pan-CT scan in picking up multiple injuries in the emergency trauma settings, justifying their liberal use. Hence, this study aims to find out the diagnostic yield of pan-CT scan in trauma patients presenting in the emergency department of a tertiary care center. This cross-sectional study has been reported in line with the STROCSS guidelines^[^8^]^.

HIGHLIGHTS

- Pan-computed tomography (pan-CT) scan was found to be effective, with positive findings in 82.1% of patients in at least one body region.
- The positivity rate was significantly higher in patients with an Injury Severity Score **≥**16.
- Despite high positivity, only 14.4% of patients needed emergency interventions, suggesting the need for careful patient selection.
- The study highlights the importance of establishing clear clinical criteria for pan-CT use in emergency trauma settings.

## Methodology

This was a cross-sectional analytical study conducted between 1 May 2023 and 30 April 2025. Ethical approval was taken from the institutional review committee of a tertiary care center. Hospital records of trauma patients presenting in the emergency department were screened. Patients who underwent pan-CT scans were included in the study. Records with missing patient information and incomplete pan-CT reports were excluded. A convenient sampling method was used. Sample size was calculated using the following formula:

Minimum required sample size *N* = z^2^xpq/e^2^

= (1.96)^2^ × 0.72 × 0.28/(0.05)^2^

= 309.78

Where, *z* = 1.96, constant for 95% confidence interval (CI)

*P* = 0.72 (72%), prevalence of positive pan-CT reports obtained from previous study^[^9^]^

*q* = 0.28, 1 – *P*

*e* = 0.05 (5%), margin of error

The calculated sample size was 310.

Demographic data, such as age, sex, mechanism of injury, and Injury Severity Score (ISS)^[^10^],^ were recorded. A CT scan was done using Siemens^®^ 64-slice scanner, and reporting was done by a team of three radiologists with more than 5 years of experience^[^11^]^. Pan-CT scan was divided into five body regions: zone 1 (head and face); zone 2 (neck and spine); zone 3 (chest); zone 4 (abdomen); and zone 5 (pelvis). The report was considered positive if any recent trauma-related injuries were identified.

Overall positivity rate (positive report of injury in at least one body region), positivity rate in 2 or more body regions, and positivity rate in 3 or more body regions were calculated. The frequency of patients requiring extra CT scans, such as contrast-enhanced CT, and those requiring emergency interventions were also recorded. Injuries identified were further classified into major (clinically significant) and minor (clinically insignificant) injuries as follows:^[^12–16^]^

Zone 1: Major injuries: intracranial hemorrhages, pneumocephalus, deep axonal injuries, and displaced skull and facial bone fractures; minor injuries: non-displaced skull and facial bone fractures and traumatic soft-tissue injuries^[^12^]^.

Zone 2: Major injuries: vertebral body fractures and dislocations; minor injuries: transverse and spinous process fractures, and traumatic soft-tissue injuries^[^13^]^.

Zone 3: Major injuries: multiple rib fractures, hemothorax, pneumothorax, alveolar hemorrhages, mediastinal injuries, and cardiac injuries; minor injuries: single rib fracture, lung contusions, and traumatic soft-tissue injuries^[^14^]^.

Zone 4: Major injuries: hemoperitoneum, pneumoperitoneum, visceral injuries, bowel perforations, and retroperitoneal hemorrhage; minor injuries: traumatic soft-tissue injuries, contusions, and minimal free fluid^[^15^]^.

Zone 5: Major injuries: visceral injuries, pelvic collection, and pelvic bone fractures with ring disruption; minor injuries: pubic rami fractures and traumatic soft-tissue injuries^[^16^]^.

The normality of the data was tested using Kolmogorov-Smirnov test. Continuous data were reported as mean ± standard deviation (SD) or median (interquartile range) based on normality. Categorical data were reported as number (percentage). Comparative analysis was carried out between patients with ISS <16 and ISS**≥**16^[^17^]^ regarding positivity rates using the Mann-Whitney *U* test and Chi-square or Fisher’s exact test. Odds ratio was also calculated for patients with ISS>16 using binary logistic regression analysis. The level of significance was set at 0.05.

## Results

A total of 313 pan-CT reports were included in the study. Out of which, 257 (82.1%) reports had positive findings in at least one body region, 166 (53.03%) had positive findings in 2 or more body regions, and 56 (17.89%) had positive findings in 3 or more body regions. Forty-five (14.4%) patients required emergency procedures, and 34 (10.9%) patients required a contrast-enhanced CT scan. Patients’ age ranged from 4 to 92 years, with a median age of 32 (24–48) years, and there were 221 (70.6%) men and 92 (29.4%) women. Mechanisms of injury were road traffic accidents (RTA) in 263 (84%) cases, high-energy fall in 34 (10.9%) cases, low-energy fall in 10 (3.2%) cases and others in 6 (1.9%) cases. ISS ranged from 6 to 48, with a median ISS of 17 (12–27). Table 1 showed positivity reports according to different body regions, and Table 2 showed the distribution of findings (Tables 1 and 2). Analysis excluding minor injuries showed that 221 (70.6%) reports had positive findings in at least one body regions, 101 (32.3%) had positive findings in two or more body regions, and 33 (10.5%) had positive findings in three or more body regions.

**Table 1:** Positivity reports according to different body regions.

Body regions	Positivity rate, *n* (%)
Zone 1 (head and face)	148 (47.3%)
Zone 2 (neck and spine)	62 (19.8%)
Zone 3 (chest)	187 (59.7%)
Zone 4 (abdomen)	43 (13.7%)
Zone 5 (pelvis)	48 (15.3%)

**Table 2:** Showing distribution of findings based on different body regions.

Body region and injury types	Frequency, *n* (%)
Zone 1	
Major injuries	103 (69.6%)
Minor injuries	46 (30.4%)
Zone 2	
Major injuries	59 (95.2%)
Minor injuries	3 (4.8%)
Zone 3	
Major injuries	121 (64.7%)
Minor injuries	66 (35.3%)
Zone 4	
Major injuries	38 (88.4%)
Minor injuries	5 (11.6%)
Zone 5	
Major injuries	37 (77.1%)
Minor injuries	11 (22.9%)

Out of 313 patients, 150 (47.9%) had ISS < 16 and 163 (52.1%) had ISS **≥** 16 (Table 3). Table 4 shows a comparison of the positivity rate between two groups, i.e., ISS < 16 and ISS **≥** 16 (Table 4).

**Table 3:** Baseline characteristics of patients with ISS < 16 and ISS ≥ 16.

Characteristics	ISS < 16 (*n* = 150)	ISS ≥ 16 (*n* = 163)	*P* value
Age in years (median, IQR)	32 (23–48)	33 (25–47)	0.423*
Gender (number, %)			
Male	102 (68)	119 (73)	0.331^#^
Female	48 (32)	44 (27)	
MOI (number, %)			
RTA	122 (81.3)	141(86.5)	0.595^#^
High energy fall	20 (13.3)	14 (8.6)	
Low energy fall	5 (3.3)	5 (3.1)	
Others	3 (2.1)	3 (1.8)	

IQR, interquartile range; SD, standard deviation; ISS, Injury Severity Score.

* Mann-Whitney *U* Test; ^#^Chi-square test.

**Table 4:** Showing comparison of positivity rates between patients with ISS ≥ 16 and ISS < 16 based on different body regions.

Body regions	Positivity rates		OR for positive findings among ISS>16 (95%CI)	*P*-value
	ISS < 16	ISS ≥ 16		
Head and face, *n* = 148	53 (35.8%)	95 (64.2%)	2.55 (1.61–4.03)	<0.001
Neck and spine, *n* = 62	29 (46.8%)	33 (53.2%)	1.05 (0.60–1.84)	0.84
Chest, *n* = 187	77 (41.2%)	110 (58.8%)	1.96 (1.24–3.11)	0.004
Abdomen, *n* = 43	17 (39.5%)	26 (60.5%)	1.48 (0.77–2.86)	0.23
Pelvis, *n* = 48	22 (45.8%)	26 (55.2%)	1.10 (0.59–2.04)	0.75

## Discussion

This study identified that the positivity rate of pan-CT scan was 82.1%, 53.03%, and 17.09% for at least one, two, and three body regions, respectively. The overall positivity rate observed in this study was higher compared to what was reported in the literature^[^3,18^]^. Ozcete *et al* (2022) conducted a study in a similar setting, including 717 patients, and found that the positivity rate was 53.7%, 23.3%, and 7.4% for at least one, two, and three body regions, respectively^[^3^]^. Similarly, Davies *et al* (2016) found the positivity rate of 58% for at least 1 body region^[^18^]^. Furthermore, Yoong *et al* (2019) found that the pan-CT had a sensitivity of 0.98 in picking up the injury in polytrauma patients^[^4^]^. High positivity rate in identifying injuries in at least 1 body region suggests higher diagnostic yield of pan-CT scan in picking up the injuries in trauma settings. However, the high positivity rate observed in this study could be due to a smaller sample size and inclusion of minor injuries, such as subcutaneous edema, scalp hematoma, muscle contusions, and minimal free fluid. After exclusion of minor injuries from the analysis, the positivity rate dropped to 70.6%, and the positivity rate in identifying injuries in two or more body regions was also low (32.3%).

In this study, only 10% required repeat scans, suggesting a major advantage of obtaining pan-CT scans in the emergency department. Some studies suggested that the pan-CT scan should be the choice of investigation for trauma screening as it helps in reducing the need for repeat scans and length of stay^[^2,17^]^. A prospective study conducted in the United States showed that the pan-CT scan protocol reduced the incidence of missed injuries by 2.7%^[^19^]^. The length of stay in the emergency room and the mean door-to-operating room time were reduced significantly^[^19^]^. However, the incidental findings were increased by 14.4%, and the cost was increased by approximately 50$ per patient. Studies conducted in the low- and middle-income countries have suggested that pan-CT possesses a high-cost burden to the patients as well as to the healthcare system^[^20,21^]^. Overutilization of CT scans also increases the total radiation exposure and estimated lifetime cancer risk^[^22,23^]^. Thus, considering increased risk of overdiagnosis, costs, and cancer risks, the utilization of pan-CT scan should be judicious.

Another utility of obtaining pan-CT scan is early identification of life-threatening injuries and subsequent management. However, in our study, only 14% of the patients required emergency interventions. See *et al* (2025), in a study involving 496 patients, reported that only 22.5% required emergency interventions^[^24^]^. Systematic and evidence-based reviews have also suggested that there is no benefit of obtaining pan-CT scans in the emergency department in terms of patient outcomes, including in-hospital mortality^[^17,25^]^. This suggests that despite having clinical significance in identifying potentially missed injuries in the emergency department, its influence on the overall patient outcome is limited.

The positivity rate of 32% for at least two body regions, excluding clinically non-significant findings, suggests overutilization of pan-CT scans in this study. This could be due to various reasons, including the desire for rapid diagnosis, the fear of missing injuries, and defensive medicine practices^[^26^]^. A systematic review has suggested that ISS of **≥**16, Glasgow Coma Scale (GCS) <13, changes in one or more vital parameters, and a high degree of suspicion could be the indication for obtaining pan-CT scan^[^17^]^. However, there is no consensus in the literature regarding the appropriate indication for obtaining pan-CT scans^[^17,25,27^]^. In this study, a subgroup analysis conducted between patients with ISS **≥** 16 and ISS < 16 suggested that the positivity rate was higher in patients with ISS **≥** 16. Odds of obtaining positive reports in the head and chest regions were around three and two times, respectively, in patients with ISS **≥** 16 than in patients with ISS < 16. This suggests that obtaining pan-CT scan would be more helpful in patients with polytrauma, involving the head and chest. However, the limitations of ISS should also be considered while applying these findings^[^10^]^.

This study has some limitations. It was a single-center study. The CT reports were reviewed retrospectively without clear information on their indication. This may increase the risk of potential selection bias. The sensitivity analysis was not done. Several confounders, such as the mechanism of injury, hemodynamic status, GCS, and changes in vital parameters, were not considered for subgroup analysis. The direct comparison between pan-CT imaging and the conventional imaging strategy was also not done. Thus, the findings can only be generalized to the hospitals in similar settings.

## Conclusion

Pan-CT scan revealed positive findings in 82.1%, 53.03%, and 17.09% in at least one, two, and three body regions, respectively. The positivity rate is significantly higher in patients with ISS **≥** 16 involving the head and chest. One tenth of the patients require a repeat scan. However, the indication of pan-CT scan in the emergency setting should be judicious, and further study should focus on establishing an appropriate indication for obtaining pan-CT scans.

## Data Availability

The datasets generated and analyzed during this study are available from the corresponding author upon reasonable request.

## Research registration unique identifying number (UIN)

researchregistry11463.
